# B-type natriuretic peptide: powerful predictor of end-stage chronic heart failure in individuals with systolic dysfunction of the systemic right ventricle

**DOI:** 10.3325/cmj.2016.57.343

**Published:** 2016-08

**Authors:** Marketa Hegarova, Jaroslav Brotanek, Milos Kubanek, Radka Kockova, Janka Franekova, Vera Lanska, Ivan Netuka, Vojtech Melenovsky, Ivan Malek, Josef Kautzner

**Affiliations:** 1 Department of Cardiology, Institute for Clinical and Experimental Medicine, Prague, Czech Republic; 2 Department of Internal Medicine, Thomayer Hospital, Prague, Czech Republic; 3 Department of Laboratory Methods, Institute for Clinical and Experimental Medicine, Prague, Czech Republic; 4 Department of Biostatistics, Institute for Clinical and Experimental Medicine, Prague, Czech Republic; 5 Department of Cardiac Surgery, Institute for Clinical and Experimental Medicine, Prague, Czech Republic

## Abstract

**Aim:**

To assess whether B-type natriuretic peptide (BNP) can serve as a predictor of end-stage chronic heart failure (CHF) in patients with severe systolic dysfunction of the systemic right ventricle (SRV).

**Methods:**

We performed a retrospective analysis in 28 patients with severe systolic dysfunction of the SRV (ejection fraction 23 ± 6%) who were evaluated as heart transplant (HTx) candidates between May 2007 and October 2014. The primary endpoints of the study (end-stage CHF) were progressive CHF, urgent HTx, and ventricular assist device (VAD) implantation. Plasma BNP levels were measured using a chemiluminescent immunoassay.

**Results:**

During median follow-up of 29 months (interquartile range, 9-50), 3 patients died of progressive CHF, 5 patients required an urgent HTx, and 6 patients underwent VAD implantation. BNP was a strong predictor of end-stage CHF (hazard ratio per 100 ng/L: 1.079, 95% confidence interval, 1.042-1.117, *P*˂0.001). The following variables with corresponding areas under the curve (AUC) were identified as the most significant predictors of end-stage CHF: BNP (AUC 1.00), New York Heart Association functional class class III or IV (AUC 0.98), decompensated CHF in the last year (AUC 0.96), and systolic dysfunction of the subpulmonal ventricle (AUC 0.96).

**Conclusion:**

BNP is a powerful predictor of end-stage CHF in individuals with systolic dysfunction of the SRV.

Transposition of the great arteries (TGA), the aorta and the pulmonary artery, is a congenital heart defect with a morphologically right ventricle pumping blood into systemic circulation. It occurs either as complete TGA (D-TGA) or congenitally corrected TGA (ccTGA). D-TGA is characterized by dextroposition of the systemic ventricle, sinistroposition of the subpulmonal morphologically left ventricle, and right-sided rotation of the great arteries. On the other hand, the term ccTGA describes sinistroposition of the the systemic ventricle, dextroposition of the subpulmonal morphologically left ventricle, and left-sided rotation of the great arteries (1). Preserved left-sided position of the pulmonary venous return and right-sided localization of the systemic venous return in both types of TGA result in almost complete separation of the systemic and pulmonary circulation in D-TGA, which must be surgically corrected in early childhood, and physiological correction of the defect in ccTGA (1). Unfortunately, both the operative correction of D-TGA by atrial tunneling (atrial switch, the Mustard or Senning procedure) and anatomic situation in ccTGA leave the morphologically right ventricle as the systemic ventricle (1-5). As compared with the morphologically left ventricle, tricuspid valve, thin walls, different ventricular shape, and myocyte architecture, make this type of the systemic ventricle susceptible to dilatation, development of tricuspid regurgitation, and heart failure (1-5).

Tricuspid regurgitation, systolic dysfunction of the SRV, and chronic heart failure (CHF) were associated with impaired survival both in individuals with D-TGA (2-4) and ccTGA (5). Prognostic stratification of these patients remains notoriously difficult, especially in the presence of SRV systolic dysfunction. Although B-type natriuretic peptide (BNP) provides strong diagnostic and prognostic information in left ventricular systolic dysfunction (6,7), its role in CHF due to SRV failure remains poorly understood. BNP and/or N-terminal pro BNP correlate just modestly with systolic SRV dysfunction (8-11). In addition, there are scarce prognostic data in individuals with SRV (11). Importantly, improved prognostic stratification could facilitate appropriate use of ventricular assist devices (VAD) and/or heart transplant (HTx) in this population (12,13). Therefore, we decided to evaluate whether BNP has a prognostic role in individuals with SRV who were referred to our heart failure clinic for consideration of HTx.

## Patients and methods

### Study population and data collection

We performed a retrospective analysis in all patients with SRV who were referred to the Heart Failure Clinic of our institution (Institute for Clinical and Experimental Medicine, Prague, Czech Republic) between May 2007 and October 2014. Available demographic, clinical, laboratory, electrocardiographic, and echocardiographic data from the first visit to our clinic were collected from the institutional database. Doses of angiotensin converting enzyme inhibitors, angiotensin receptor blockers, and beta-blockers were expressed as a percentage of the maximum recommended daily dose according to the latest European Society of Cardiology guidelines (14). The patients were followed until July 30, 2015. Vital status and the necessity of urgent HTx or VAD implantation were recorded. The primary endpoint of this study included death, urgent HTx, or VAD implantation. It was considered an equivalent of end-stage CHF.

The study group consisted of 28 consecutive patients with SRV. This group included 21 patients (75%) after atrial switch due to D-TGA (14 patients with the Mustard procedure, 7 patients with the Senning procedure) and 7 patients (25%) with ccTGA. The following associated lesions were corrected during the primary cardiac surgery: ventricular septal defect in 6 patients with atrial switch, ventricular and atrial septal defect in 1 patient with ccTGA, and ventricular septal defect and valvular pulmonary artery stenosis in another patient with ccTGA. 5 patients had a history of reoperation: 3 patients with atrial switch and 1 patient with ccTGA required tricuspid valve replacement, while additional 1 patient after atrial switch underwent aortic valve replacement due to severe regurgitation. All 20 men (71%) and 8 women (29%) with mean age of 34 years (range 15-65) had at the first evaluation severe systolic SRV dysfunction with mean ejection fraction of 23 ± 6% ranging from 14% to 35%. Median BNP plasma level was 618 ng/L (interquartile range [IQR] 86.5-1363.5) (Table 1). This study conformed to the principles of the Declaration of Helsinki. It was approved by the ethics committee of the Institute for Clinical and Experimental Medicine, Prague, Czech Republic.

**Figure 1:**
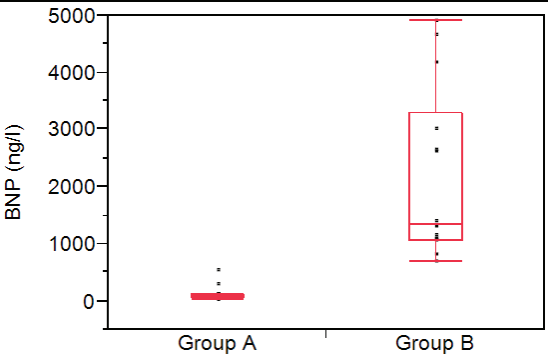
Comparison of plasma levels of B-type natriuretic peptide (BNP) in the group A (favorable outcome) and group B (unfavorable outcome). Data are shown as a boxplot.

### Laboratory methods

Blood samples for the measurement of BNP plasma levels were collected into chilled tubes containing EDTA (1 mg/mL) and aprotinin (500 kallikrein inactivator U/mL). Test tubes were immediately centrifuged. Plasma BNP levels were measured using a chemiluminescent immunoassay (Abbott Laboratories, Diagnostics Division, Abbott Park, IL, USA, Supplier: Abbott, Max-Planck-Ring 2, Wiesbaden, Germany). The lower limit of detection was 10 ng/L, intra-assay coefficient of variation (CV)<3.8%, and inter-assay CV<5.3%. For serum creatinine assessment, we used an enzymatic method (Abbott Architect Creatinine, catalog No. 8L24-31, Abbott Laboratories Inc.).

### Echocardiography

Echocardiography was performed by experienced operators in accordance with the current guidelines of the American Society of Echocardiography (15,16). Standardized echocardiographic parasternal and apical views were performed on Vivid 7 and Vivid E9 (GE HealthCare, Horten, Norway) ultrasound system equipped with a 2-dimensional (2D) probe. Parasternal and apical 2- and 4- chamber and long axis views were acquired with the high frame rate and full image optimization (depth, sector size, gain). All acquired images were stored and analyzed off-line using commercially available software (EchoPac BT 12, GE HealthCare). The anatomically right ventricular (RV) end-diastolic diameter was measured in RV-focused apical 4-chamber view at the basal level. RV volumes and systolic function were measured using fractional area change (FAC). The anatomically left ventricular (LV) end-diastolic diameter was measured in the long axis parasternal view. Biplane disk summation method was used for LV volumes and ejection function assessment (LVEF). The following grading of LV systolic dysfunction was used: none (LVEF>55%), mild (LVEF 46%-55%), moderate (LVEF 36%-45%), and severe (LVEF≤35%). Atrioventricular valve regurgitation was assessed semiquantitatively using Doppler methods (color, pulse, continuous wave Doppler). Pulmonary artery pressure was calculated based on continuous Doppler measurement across the left atrioventricular valve with the addition of estimated right atrial pressure based on the inferior vena cava diameter and collapsibility.

### Statistical analysis

Categorical data were described using absolute and relative frequencies, and compared using χ^2^ analysis or Fisher exact test. Continuous variables were assessed for normality using the Kolmogorov-Smirnov test. Normally distributed continuous variables are shown as a mean and standard deviation. Not-normally distributed continuous variables are shown as a median and interquartile range. Continuous variables were compared using the *t* test for unpaired data or non-parametric Mann-Whitney test where appropriate. Univariate Cox regression models were used to identify the predictors of the primary endpoint. Kaplan-Meier curves were constructed to illustrate the relationship between selected variables and time-dependent events. Due to limited sample size and number of events, we could not apply multivariate Cox regression analysis. Receiver operating characteristic curve (ROC) analysis and Youden’s index (sensitivity + specificity – 100) were used to assess and compare the performance of selected variables to predict the primary endpoint. For all tests a probability value of *P* < 0.050 was considered significant. All analyses were performed using the statistical software SPSS, version 17.0 (SPSS Inc., Chicago, IL, USA).

## Results

### Events during the follow-up

During a median follow-up of 29 months (IQR 9-50), 14 patients experienced the primary endpoint. 3 patients (11%) died of end-stage HF after 5, 14, and 15 months. 5 patients (18%) required an urgent HTx (after 4-14 months) and 6 patients (21%) underwent VAD implantation (HeartMateII, Thoratec Corporation, Pleasanton, CA, USA) after 0-17 months as a bridge to transplant. The remaining 14 individuals were free of the primary endpoint (group A, favorable outcome), whereas the above mentioned 14 patients were included in the group B (unfavorable outcome).

### Predictors of the unfavorable outcome

**Figure 2:**
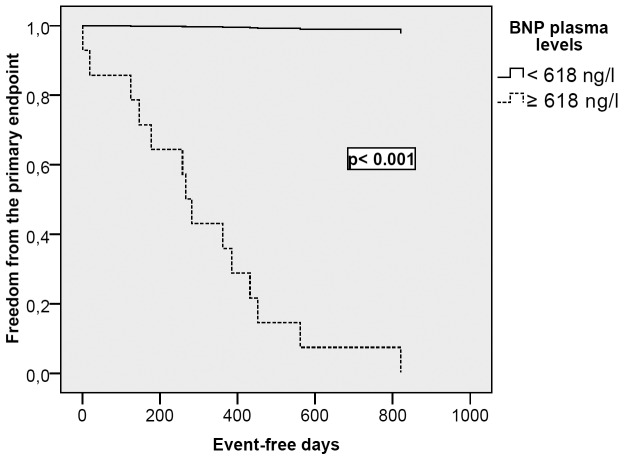
Kaplan-Meier curve illustrates survival without the necessity of urgent heart transplantation or ventricular assist device implantation according to median of B-type natriuretic peptide (BNP) plasma levels (median = 618 ng/L).

Unfavorable outcome in group B was predicted by the following signs of advanced CHF: a history of decompensated HF in the last 12 months, higher New York Heart Association (NYHA) functional class, higher heart rate, lower systolic blood pressure, markedly elevated plasma levels of BNP (Figure 1), higher creatinine serum levels, worse tolerance of angiotensin converting enzyme inhibitors or sartans, and higher daily doses of furosemide. Advanced stage of cardiac remodeling in group B correlated with a broader QRS complex on ECG, lower ejection fraction of the SRV, higher occurrence of severe regurgitation on the systemic atrioventricular valve, and the presence of systolic dysfunction of the subpulmonal ventricle. Similar results were revealed using univariate Cox regression analysis (Table 2). Efficacy of clinically relevant predictors of the unfavorable outcome was compared using the ROC curve analysis (Table 3). The best area under the curve (1.00) and the Youden’s index (100%) was found for BNP (optimal cutpoint of 618 ng/L), followed by clinical parameters such as NYHA class, a history of decompensated CHF, and systolic dysfunction of the subpulmonal ventricle. Other variables had a suboptimal predictive capacity (Table 3). Figure 2 illustrates significant separation of Kaplan-Meier curves in individuals with BNP plasma levels above and below the median.

**Table 2:** Results of univariate Cox regression analysis. Predictors of death, urgent heart transplantation, or ventricular assist device implantation*

Variables	χ^2^	Hazard ratio (95% confidence interval)	*P*
History of decompensated heart failure in the last 12 mo	25.20	162.20 (1.57-1649.70)	<0.001
NYHA functional class (per 1 class)	14.89	16.95 (4.030-71.350)	<0.001
Heart rate (per 10 beats/min)	12.51	1.783 (1.284-2.476)	<0.001
Systolic blood pressure (per 1 mm Hg)	3.92	0.950 (0.903-1.000)	0.048^†^
QRS duration (per 10 ms)	12.07	1.403 (1.146-1.719)	0.001^‡^
Serum sodium (per 1 mmol/L)	6.19	0.776 (0.634-0.949)	0.013^†^
Serum creatinine (per 10 µmol/L)	29.34	1.347 (1.061-1.710)	<0.001
Glomerular filtration rate (per 10 mL/min)	2.68	0.871 (0.736-1.030)	0.101
B-type natriuretic peptide (per 100 ng/L)	29.21	1.079 (1.042-1.117)	<0.001
Ejection fraction of the SRV (per 1 percent point)	8.58	0.844 (0.748-0.951)	0.003^‡^
Severe regurgitation on the systemic atrioventricular valve	7.20	4.98 (1.37-18.14)	0.007^‡^
Any systolic dysfunction of the subpulmonal ventricle	32.74	2.76 (1.67-4.27)	<0.001
Treatment with ACEi/sartan	16.57	0.111 (0.031-0.395)	<0.001
Treatment with furosemide	5.51	7.862 (1.024-60.34)	0.008^‡^
Daily dose of furosemide (per 10 mg/d)	32.62	1.258 (1.097-1.441)	<0.001

*ACEi – angiotensin converting enzyme inhibitor; SRV – systemic right ventricle; NYHA – New York Heart Association.

†*P*-value was coded as *P* < 0.05.

‡*P*-value was coded as *P* < 0.01.

**Table 3:** Results of the receiver operating characteristic curve analysis. Selected variables are listed according their accuracy to predict the combined endpoint (death, urgent heart transplantation, or ventricular assist device implantation)*

Variable	AUC	Optimal cutpoint	Youden's index
B- type natriuretic peptide (ng/L)	1.00	618	100.0
NYHA functional class	0.98	III	92.9
History of decompensated heart failure in the last year	0.96	yes	92.9
Systolic dysfunction of the subpulmonal ventricle	0.96	yes	92.9
Heart rate (beats per min)	0.83	68	47.2
QRS duration (ms)	0.81	139	62.6
Creatinine (µmol/L)	0.80	90	57.1
Severe tricuspid regurgitation	0.74	yes	47.8
Treatment with furosemide	0.69	yes	39.1

*AUC – area under the curve, NYHA – New York Heart Association, SRV – systemic right ventricle.

## Discussion

To the best of our knowledge, this is the largest clinical study that evaluated the prognostic value of BNP in individuals with severe systolic dysfunction of the SRV. First, BNP was found to be a powerful predictor of end-stage CHF in individuals with systolic dysfunction of the SRV. Second, NYHA functional class III or IV and a history of decompensated CHF, well-known predictors of adverse outcome in patients with SRV, and any dysfunction of the subpulmonal ventricle provided additional important prognostic information.

Systolic SRV dysfunction limits the long-term prognosis both in individuals with atrial correction of D-TGA and ccTGA who frequently die of CHF or malignant arrhythmias. Recent studies demonstrated a cumulative survival after Mustard correction of D-TGA in the range of 80% and 68% at 20 and 39 years, respectively (3,17). Another study (18) identified CHF in approximately 20% of individuals after atrial switch due to D-TGA and in 30% of patients with ccTGA. The most important risk factors of CHF were NYHA functional class and systolic dysfunction of the SRV. Available studies demonstrated just a modest correlation of BNP and/or N-terminal proBNP with the functional status and systolic function of the SRV (8-11,19). In addition, another two reports found a correlation between worsening of systolic function of the SRV and increasing levels of N-terminal proBNP (20,21). However, just two studies were prognostic in nature (11,22). The study of Larson et al (11) was underpowered, with 21 patients with systolic dysfunction of the SRV (ejection fraction ≤45%) and 1 death during the follow-up. Not surprisingly, this study failed to detect prognostic relevance of natriuretic peptides. On the other hand, in a study of Haberer et al (22), BNP higher than 85 ng/L predicted 1 death, necessity of HTx in 3 patients, and hospitalization due to decompensated CHF in 8 patients. These patients were classified as NYHA class I and II (91%) with missing echocardiographic data. On the contrary, more than 50% of patients in our study group were in NYHA class III or IV and they had severe systolic dysfunction of the SRV (ejection fraction ≤35%).

Although most patients with systolic dysfunction of the SRV receive conventional pharmacotherapy for CHF, it should be noted that in this population beta-blockers, angiotensin converting enzyme inhibitors, and angiotensin receptor blockers showed mixed results (23-27). Management of adult patients with severe systolic dysfunction of the SRV is difficult, because even stable patients in NYHA functional class II may progress very quickly to end-stage CHF and require advanced non-pharmacological methods. On the other hand, congenital heart disease has been identified as an important risk factor for early mortality after HTx. However, the long-term survival of these individuals is satisfactory due to young age and few comorbidities (12,13). Early mortality may be increased due to complicated anatomic situation or sequalae after previous corrective cardiac surgery or VAD implantation. These complications include tissue adhesions, disappearance of the pericardial space, and allosensitization with resulting risk of antibody-mediated rejection. In addition, patients with congenital heart disease may spend longer time on the waiting list due to specific demands on donors and availability of experienced surgeons (28-32). Some authors suggest early enlistment of patients with complex congenital heart disease for heart transplant to prevent VAD implantation and reduce the number of reoperations (31). BNP measurement might facilitate risk stratification in patients with failing SRV and could improve the timing of non-pharmacological treatment.

There are several limitations of this study. First, the limited number of patients and retrospective study design that reflect the rare occurrence of CHF due to SRV failure may limit general applicability of the study results. A larger study, perhaps a multicenter one, might increase the strength of our findings. However, relatively high mortality and morbidity of the studied population allowed even prognostic assessment. Second, limited number of events precluded the use of multivariate models. Already two variables in one model would result in the risk of “overfitting” (33). Finally, the selected cut-off points predicting the study end-point have to be considered specific for the studied population and may vary in different populations.

In conclusion, BNP is a powerful predictor of end-stage CHF in individuals with congenital heart disease and systolic dysfunction of the SRV. NYHA class III or IV, a history of decompensated CHF, and any systolic dysfunction of the subpulmonal ventricle provided important additional prognostic information. These findings might facilitate prognostic stratification in patients with CHF due to the SRV failure and could improve the timing of heart transplant enlistment or VAD implantation.

## 

**Table 1:** Characteristics of the group with the favorable outcome and the group with an unfavorable outcome (death, urgent heart transplantation, ventricular assist device implantation)*

	Favorable course (group A) N = 14	Unfavorable course (group B) N = 14	*P*
Age (years), mean ± standard deviation	36.4 ± 15.3	31.5 ± 7.9	0.302
Sex (male/female), n (%)	8 (57)/6 (43)	12 (86)/2 (14)	0.209
Body mass index (kg/m^2^), mean ± standard deviation	23.9 ± 4.1	23.4 ± 3.3	0.306
TGA with atrial switch, n (%)	10 (71)	11 (79)	1.0
Type of atrial switch Mustard/Senning, n (%)	5 (36)/5 (36)	9 (64)/2 (14)	0.276
Congenitally corrected TGA, n (%)	4 (29)	3 (21)	1.0
Associated lesions corrected at the primary operation, n (%)	5 (36)	3 (20)	0.678
Reoperation- tricuspid valve replacement, n (%)	3 (21)	1 (7)	0.596
Reoperation- aortic valve replacement, n (%)	0	1 (7)	1.0
Implantable cardioverter-defibrillator, n (%)	4 (29)	6 (43)	0.695
History of decompensated heart failure in the last 12 mo, n (%)	1 (7)	14 (100)	<0.001
NYHA functional class, n (%)			<0.001
I	5 (36)	0	
II	8 (57)	0	
III	1 (7)	8 (57)	
IV	0	6 (43)	
Heart rate (beats/min), mean ± standard deviation	64 ± 19	83 ± 18	0.006^†^
Systolic blood pressure (mm Hg), mean ± standard deviation	117 ± 9	108 ± 14	0.016^‡^
Rhythm, n (%)			0.092
sinus	12 (86)	9 (64)	
atrial fibrillation	2 (14)	1 (7)	
paced	0	4 (29)	
QRS duration (ms), mean ± standard deviation	121 ± 27	157 ± 32	0.004^†^
Serum sodium (mmol/L)	138.0 ± 2.0	136.2 ± 4.2	0.166
Serum creatinine (µmol/L), mean (interquartile range)	77 (66-87)	97 (81-113)	0.005^†^
Glomerular filtration rate (mL/min), mean ± standard deviation	122 ± 41	101 ± 39	0.186
B-type natriuretic peptide (ng/L), mean (interquartile range)	91 (64-173)	1342 (1071-3301)	<0.001
End-diastolic diameter of the SRV (mm), mean ± standard deviation	53 ± 12	59 ± 16	0.346
Ejection fraction of the SRV (%), mean ± standard deviation	26.4 ± 5.7	19.2 ± 3.4	0.001^†^
Regurgitation on the systemic atrioventricular valve, n (%)			0.037^‡^
mild	4 (29)	0	
moderate	3(21)	2 (14)	
severe	4(29)	11 (79)	
replaced	3 (21)	1(7)	
Systolic dysfunction of the subpulmonal ventricle, n (%)			<0.001
none	14 (100)	1 (7)	
mild	0	3 (21)	
moderate	0	4 (29)	
severe	0	6 (43)	
Treatment with ACEi/sartan, n (%)	14 (100)	9 (64)	0.041^‡^
Equivalent dose of ACEi/sartan ≥50% of recommended, n (%)	6 (43)	3 (21)	0.420
Treatment with a beta-blocker, n (%)	9 (64)	10 (71)	1.00
Equivalent dose of the beta-blocker ≥50% of recommended, n (%)	4 (29)	5 (36)	1.00
Treatment with digoxin, n (%)	2 (14)	6 (43)	0.209
Treatment with furosemide, n (%)	7 (50)	13 (93)	0.033^‡^
Daily dose of furosemide (mg/d), median (interquartile range)	5 (0-75)	50 (40-65)	0.001^†^
Mineralocorticoid receptor blocker, n (%)	6 (43)	11 (79)	0.120

*ACEi – angiotensin converting enzyme inhibitor; SRV – systemic right ventricle; TGA – transposition of great arteries; NYHA – New York Heart Association.

†*P*-value was coded as *P* < 0.01.

‡*P*-value was coded as *P* < 0.05.
